# Le lymphomes non hodgkinien primitif de la thyroïde: à propos de sept cas

**Published:** 2012-07-19

**Authors:** Hanane Chenna, Hanane Berhil, Karima Nouni, Hanane Kabbaj, Hanane Zaidi, Ahmedou Toulba, Hanan El Kacemi, Khalid Hassouni, Tayeb Kebdani, Brahim Khalil El Gueddari, Noureddine Benjaafar

**Affiliations:** 1 Service de radiothérapie, institut national d'oncologie de Rabat, BP 6213 Rabat, Maroc

**Keywords:** Lymphome primitif de la thyroïde, traitement, Primtive lymphoma, thyroid, treatment

## Abstract

Les lymphomes non hodgkiniens primitifs de la thyroïde sont rares : ils représentent moins de 2 à 5 % des cancers de la thyroïde. L'objectif de ce travail est de revoir les stratégies diagnostiques et thérapeutiques actuelles des lymphomes non hodgkiniens primitifs de la thyroïde tout en présentant notre expérience à l'Institut national d'oncologie. Nous rapportons à travers une étude rétrospective, sept cas des lymphomes non hodgkiniens primitifs de la thyroïde colligés à l'Institut National d'Oncologie au Maroc entre 2004 et 2008. Sept patients ont été inclus dans notre étude, l’âge médian au moment du diagnostic était de 50 ans, avec sexe ratio de 2.5 à prédominance féminine. Le diagnostic histopathologique avec l'immunomarquage après chirurgie a conclu dans tous les cas à un lymphome malin non hodgkinien : 3 patientes avec un lymphome de type MALT, et les 4 autres avec un LMNH à grandes cellules de phénotype B. Une thyroïdite chronique lymphocytaire d'Hashimoto coexistante avec le lymphome malin primitif de la thyroïde était retrouvée chez une patiente. Concernant le traitement du lymphome, 5 patientes ont reçu une polychimiothérapie, et les 2 autres ont bénéficié d'un traitement combiné comportant la chimiothérapie et la radiothérapie. Une patiente est décédée 3 mois après la fin du traitement. Le suivi médian des autres patients était de 24 mois et on ne notait aucune récidive de lymphome. Les lymphomes non hodgkiniens primitifs de la thyroïde sont rares. Le traitement combiné par une chimiothérapie et radiothérapie a prouvé son efficacité. Le pronostic des stades localisés est généralement favorable.

## Introduction

Les lymphomes non hodgkiniens primitifs de la thyroïde sont rares : ils représentent moins de 2 à 5 % des cancers de la thyroïde. Ils affectent essentiellement les sujets âgés. Une thyroïdite lymphocytaire est souvent associée, expliquant en partie la prédominance féminine de ce lymphome. Différents types histologiques sont individualisés mais les plus fréquents sont les lymphomes B diffus à grandes cellules et les lymphomes de MALT. La chimiothérapie et/ou la radiothérapie représentent actuellement les meilleurs choix, la chirurgie n'étant utile que dans un but diagnostique et non thérapeutique. Nous rapportons sept cas de lymphomes thyroïdiens tout en précisant les particularités épidémiologiques, diagnostiques, thérapeutiques et pronostiques de ces tumeurs.

## Méthodes

Nous avons mené une étude rétrospective à l'Institut National d'Oncologie de Rabat au Maroc sur une période de cinq ans (2004 - 2008). Nous avons revu tous les dossiers de patients porteurs de lymphomes non hodgkinien primitif de la thyroïde certifiées histologiquement. Ont été exclus tous les dossiers de diagnostic douteux. Une fiche d'exploitation a permis de relever les principales caractéristiques épidémiologiques (sexe, âge, antécédents personnels et familiaux), cliniques (délai et motif de consultation : tuméfaction cervicale, adénopathies cervicales, signes compressifs), paracliniques (aspects à l’échographie et à la scintigraphie thyroïdienne, bilan d'extension locorégional et des bilans biologiques avec dosages des hormones thyroïdiennes), histologiques (moyens de confirmation diagnostique et type histologique), thérapeutiques (chirurgie, radiothérapie, chimiothérapie et traitement hormonal substitutif) et évolutives (recul, survenue de récidives locales ou métastatiques, décès).

## Résultats

Il s'agit d'une étude rétrospective concernant sept observations cliniques des patients atteints d'un lymphome non hodgkinien primitif de la thyroïde traités à l'Institut national d'oncologie de Rabat durant une période de cinq ans (2004-2008). La médiane d’âge de nos patients était de 50 ans avec sexe ratio de 2.5 à prédominance féminine. Dans tous les cas, la palpation thyroïdienne était anormale au moment du diagnostic, dans un contexte de tuméfaction cervicale d'apparition récente, avec la présence des signes compressifs dans 5 cas. Une échographie thyroïdienne réalisée chez tous les patients a révélé un nodule hypoéchogène chez deux d'entre eux, et un goitre hétérogène chez les sept autres, avec présence d'adénopathies cervicales chez deux cas. La thyroïdectomie totale a été réalisée chez trois patientes alors que les quatre autres ont bénéficié d'une isthmolobectomie. L'examen histopathologique avec immunomarquage a conclu dans tous les cas un lymphome malin non hodgkinien : 3 patientes avec un lymphome de type MALT, et les 4 autres avec un LMNH à grandes cellules de phénotype B. Une thyroïdite chronique lymphocytaire d'Hashimoto coexistait avec le lymphome malin primitif de la thyroïde et a été retrouvée chez une patiente. Concernant le traitement adjuvant, 5 patientes ont reçu une polychimiothérapie et les 2 autres ont bénéficié d'un traitement combiné complémentaire comportant la chimiothérapie et la radiothérapie sur le site thyroïdien et les aires ganglionnaires cervicales. Une patiente est décédée 3 mois après la fin du traitement. Le suivi médian des autres patients était de 24 mois et on ne notait aucune récidive de lymphome.

## Discussion

Le lymphome malin primitif de la thyroïde est une tumeur rare. L’âge d'apparition de cette tumeur est souvent de plus de 60 ans [1], cependant dans notre série, on a décrit des cas de lymphome malin primitif de la thyroïde chez des personnes âgées de moins de 60 ans. La symptomatologie clinique est dominée par l'apparition d'un nodule ou d'une masse thyroïdienne rapidement évolutive avec des signes de compression des organes de voisinage. Alors que les signes cliniques de dysthyroïdie sont rarement présents [2, 3]. Aozasa et al. ont montré la présence des lésions de thyroïdite chronique à l'examen histopathologique de la glande chez 83 % des patients ayant un lymphome malin primitif de la thyroïde [4]. A l’état actuel, l'association lymphome malin primitif de la thyroïde et thyroïdite chronique est bien connue [5, 6]. Cette dernière est considérée comme un état prélymphomateux et le risque de survenue d'un lymphome malin primitif de la thyroïde est multiplié par 67 chez les patients porteurs d'une thyroïdite autoimmune [7]. Le développement d'un lymphome in situ survient dans 1,5 à 5 % des cas [7, 8]. Concernant notre série, une seule patiente avait des signes histologiques de thyroïdite chronique.

Le diagnostic de lymphome malin primitif de la thyroïde repose essentiellement sur l’étude anatomopathologique après la réalisation d'une biopsie chirurgicale qui permet l'obtention d'un prélèvement de meilleure qualité. La cytoponction à l'aiguille fine n'a aucune valeur diagnostique [9] et permet essentiellement d'exclure les nodules bénins alors que la biopsie percutanée de complément est réfutée par plusieurs auteurs, car les artéfacts traumatiques provoqués par l'introduction du trocart perturbent l'interprétation de l'infiltrat lymphocytaire. Sur le plan thérapeutique, Le traitement du lymphome malin primitif de la thyroïde n'est pas clairement établi. Le rôle de la chirurgie a longtemps été débattu [10]. Le recours à la chirurgie est utile en présence de compression et/ou en cas de diagnostic histopathologique difficile, mais l'exérèse chirurgicale n'a pas démontré qu'elle améliorait le pronostic [11]. Friedberg et al. estiment que le geste chirurgical permet de distinguer les lymphomes malins primitifs de la thyroïde localisés à la glande de ceux ayant une extension extracapsulaire [10]. Dans le premier cas, cette équipe préconise la radiothérapie cervico-médiastinale comme traitement de choix. En cas d'extension extracapsulaire, un traitement combiné par chimiothérapie et radiothérapie est recommandé. D'autres attitudes thérapeutiques ont été proposées. Dans une étude concernant 31 patients, avec lymphome malin primitif de la thyroïde de type MALT de stades I et II, le taux de survie à cinq ans était de 90 % après radiothérapie exclusive [12]. La chimiothérapie seule a aussi fait preuve d'efficacité en cas de lymphome malin primitif de la thyroïde de stade IE et IIE. Leedman et al. ont rapporté trois cas de rémission complète, 26 à 36 mois après ce traitement [13]. Une étude rétrospective concernant 119 patients, a montré que le taux de survie à huit ans était de 100 % après un traitement associant six cures d'une polychimiothérapie et une radiothérapie, alors qu'il était de 75 % chez les patients qui n'avaient reçu qu'une à deux cures de chimiothérapie [2]. Dans notre série, après la chirurgie 5 patientes ont reçu la polychimiothérapie, et les 2 autres ont bénéficié d'un traitement combiné. Une patiente est décédée 3 mois après la fin du traitement. Le suivi moyen des autres patients était de 24 mois et on ne notait aucune récidive de lymphome. Au total, le traitement combiné par une chimiothérapie et radiothérapie semble la meilleure attitude thérapeutique, et le pronostic des stades localisés reste généralement favorable [14].

## Conclusion

Par leur rareté et leur polymorphisme clinique, les lymphomes de la thyroïde posent encore des difficultés diagnostiques. La conduite thérapeutique est actuellement mieux codifiée. La surveillance, en dehors du traitement hormonal substitutif, est celle de tous les lymphomes. Leur pronostic, dépend comme tout lymphome de l'histologie et du stade de la maladie, est généralement favorable avec un taux de survie à 5 ans de 70 à 80 %.
